# A domain knowledge-enhanced lightweight transformer for automatic identification of land-use conflicts in territorial spatial planning texts

**DOI:** 10.1371/journal.pone.0359870

**Published:** 2026-10-01

**Authors:** Feng Yang, Miaoxing Zhao, Leixiang Ding, Yan Li, Jing Wang

**Affiliations:** Henan University of Urban Construction, Pingdingshan, Henan, China; Philadelphia University, JORDAN

## Abstract

Implicit information on land use conflicts in territorial spatial planning texts directly affects the coordination and implementability of planning schemes. Traditional manual interpretation approaches have low efficiency, high omission rates, and inadequate standardization, making them difficult to use for full-process review of massive multi-source planning texts under multi-plan integration. To address this problem, this study proposes a lightweight Transformer-based framework for automatic land use conflict identification by integrating domain prior features with deep semantic encoding. Multi-type texts related to territorial planning were collected from official and authoritative channels to construct a domain-specific dataset containing 1200 valid samples. A multi-dimensional feature system with strong discriminability and interpretability was then extracted by combining professional knowledge of territorial planning with text statistical attributes. Finally, a lightweight Transformer model with a total parameter count of only 700000 was designed. Through the deep fusion of textual semantic features and statistical features, the model performs binary classification of land use conflicts. Experimental results show that the proposed model achieved an accuracy of 98.75% and an F1-score of 96.9% on the held-out test set, with an inference time of less than 0.5 seconds for a single text. On the 240-sample held-out test set, the 95% confidence interval for accuracy was 96.39–99.57%, and bootstrap resampling gave a 95% interval of 92.93–100.00% for the F1-score, indicating that the point estimates were not driven by a small number of individual cases. The framework may support lightweight local deployment in grassroots planning management departments after site-specific validation and can provide an auxiliary tool for planning compilation, compliance review, and ex-ante early warning of conflicts in territorial spatial planning.

## 1 Introduction

With the continuous deepening of the reform of multi-plan integration in China, territorial spatial planning has become the core statutory basis for coordinating whole-territory land use and balancing ecological protection with urban-rural development. It serves as a guideline for national spatial development and as a spatial blueprint for sustainable development [1]. The 14th Five-Year Plan for the Protection and Utilization of Natural Resources explicitly proposes improving the digital and intelligent level of territorial spatial governance and strengthening compliance review and contradiction investigation throughout the planning process. These requirements place higher demands on the efficiency and accuracy of conflict identification in planning texts.

Land use conflicts among plans at different levels and in different fields constitute a core bottleneck restricting the implementation of planning schemes. Such conflicts include explicit contradictions in planning indicators and land use boundaries, as well as implicit land use disputes embedded in policy documents, implementation cases, and public opinion reports. These implicit conflicts are difficult to cover fully through traditional review models [2]. Rapid and accurate identification of land use conflict hotspots from massive and heterogeneous textual data has therefore become a core technical demand for improving territorial spatial planning management and control [3–8].

Traditional land use conflict identification and planning review rely heavily on manual interpretation by domain experts. This process entails high labor costs and long review cycles, and it is susceptible to variation in the experience of human inspectors. As a result, key contradictions may be omitted and judgment criteria may become inconsistent, making manual review insufficient for regular and full-scale text screening. In recent years, natural language processing (NLP) technology has provided a mature technical approach for intelligent mining of text-based planning information, and Transformer-based pre-trained language models have shown strong contextual modeling capability in text classification and semantic understanding tasks [9]. However, mainstream large-parameter models, such as BERT-base, have hundreds of millions of parameters, require large amounts of annotated data, and impose high hardware thresholds for deployment. These constraints may limit their practical use in grassroots planning departments with limited computing resources and scarce annotated samples [10–14].

To address these application limitations [15–21], this study focuses on land use conflict identification in territorial spatial planning texts and constructs a domain-specific text dataset for this field. Leveraging expert domain knowledge, this study establishes a specialized keyword dictionary for land use conflicts and designs a lightweight Transformer architecture with only 700000 parameters. Through multi-dimensional fusion of semantic and statistical features, the framework improves classification performance on the constructed domain-specific dataset. The effectiveness and robustness of the proposed model are evaluated through comparative experiments, repeated stratified cross-validation, and ablation studies. The overall architecture of the end-to-end framework is illustrated in Fig 1.

**Fig 1:**
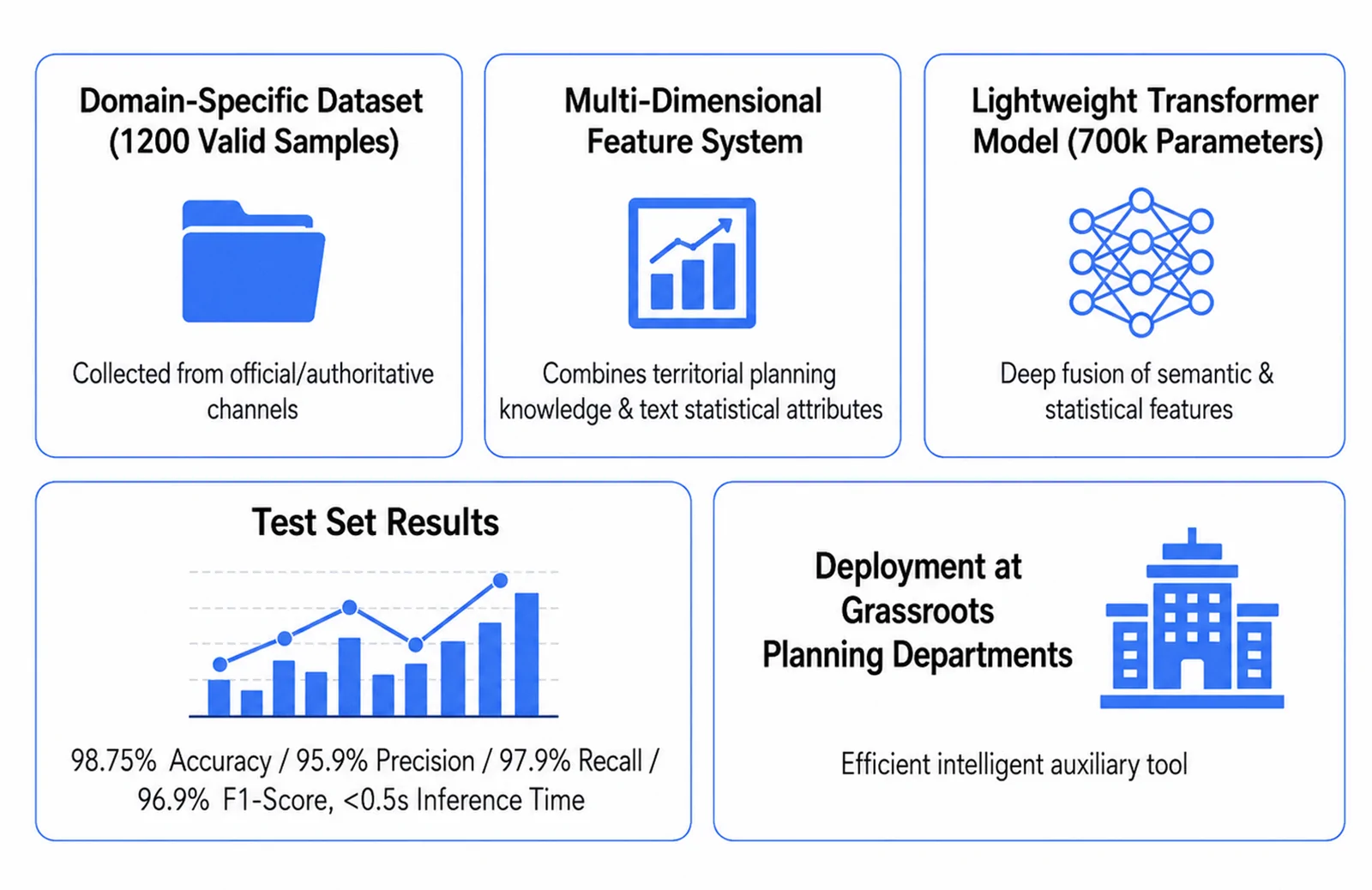
Framework of lightweight Transformer-based land use conflict identification.

While Large Language Models (LLMs) such as ChatGPT have demonstrated broad text comprehension, their direct application in grassroots planning departments faces practical barriers. First, planning conflict dossiers often contain confidential spatial and administrative information, limiting API-based cloud uploads under data-security requirements. Second, local natural resource bureaus typically lack the GPU hardware required to run billion-parameter open-source LLMs locally. Third, general-purpose LLMs may produce unsupported outputs and may not consistently follow specialized territorial spatial planning terminology, such as “Three Zones and Three Lines”. Therefore, the proposed 700000-parameter lightweight model, augmented with expert-knowledge rules, provides an interpretable and locally deployable option for government use.

The remainder of this article is organized as follows. Section 2 reviews studies on land-use conflict identification, NLP-assisted planning analysis, and lightweight feature-fusion models. Section 3 describes the dataset, preprocessing procedure, domain feature system, model architecture, experimental configuration, and statistical reliability analysis. Section 4 reports baseline performance, comparative experiments, uncertainty estimates, ablation evidence, and practical case validation. Section 5 summarizes the main findings, potential applications, and limitations of the proposed framework.

## 2 Related work

### 2.1 Traditional approaches to land-use conflict identification

Traditionally, the identification of land-use conflicts has relied on spatial overlay analysis using Geographic Information Systems (GIS) and manual review of planning documents. GIS is effective for identifying explicit boundary overlaps, but it does not capture implicit contradictions hidden within policy texts, stakeholder meeting minutes, and legal compliance reports. Manual document review remains the primary method for extracting such implicit conflicts; however, it is subjective, labor-intensive, and difficult to scale to the volume of multi-source texts generated during the modern multi-plan integration era.

### 2.2 Application of NLP in urban planning

With the rapid advancement of NLP, researchers have begun employing text mining techniques to extract spatial intelligence from unstructured data. Early studies primarily utilized traditional machine learning algorithms, including SVM and Random Forest, coupled with TF-IDF or Word2Vec embeddings for urban sentiment analysis and policy classification. Recently, deep learning models, particularly Transformers such as BERT and RoBERTa, have achieved competitive performance in spatial text classification. However, these generalized pre-trained models often struggle to capture the specialized vocabulary of Chinese territorial spatial planning without extensive domain-specific fine-tuning.

### 2.3 Lightweight models and feature fusion

A major bottleneck in applying advanced deep learning to specific administrative domains is the lack of large annotated datasets and the computational constraints of edge deployments. To address data scarcity, researchers have proposed fusing domain-specific knowledge graphs or expert lexicons with neural networks. This hybrid approach allows models to leverage explicit prior knowledge and reduces reliance on massive training corpora. Inspired by these studies, this study designs a lightweight architecture that fuses statistical text features and a specialized land-use conflict lexicon with Transformer-based semantic embeddings, aiming to balance accuracy, interpretability, and model size under resource-constrained settings.

Table 1 compares representative studies published in 2025–2026 by data type, methodological advantage, and remaining limitation. Recent land-use and remote-sensing studies have improved spatial simulation, segmentation, and change detection, but their datasets mainly consist of raster images, spatial grids, or land-cover products rather than administrative planning texts. The present study therefore focuses on text-based land-use conflict identification and combines a domain keyword lexicon with a lightweight Transformer to screen implicit conflicts under limited local computing resources.

**Table 1:** Comparison with recent land-use and AI studies published in 2025–2026.

Study	Dataset or task	Main advantage	Limitation and relation to this study
Wang et al. [2]	Regional land-use transition data	Reveals spatiotemporal land-use dynamics	Does not identify implicit conflict semantics in planning text
Wan et al. [4]	Urban expansion assessment	Uses deep models for planning-related assessment	Targets urban expansion rather than policy-document conflicts
Wassan et al. [5]	Rural land-cover segmentation	Improves visual feature extraction	Requires image data and cannot process administrative text
Shu et al. [6]	Real-time land-cover mapping	Emphasizes lightweight deployment	Solves pixel-level segmentation rather than document classification
Kussul et al. [11]	Land-use change digital twin	Integrates AI with spatial decision support	Focuses on land-use change, not planning-text review
AbdElhamied et al. [13]	Building-change detection	Addresses government-land encroachment from imagery	Cannot capture policy contradictions expressed only in text
This study	1200 planning texts	Fuses semantic, keyword, and statistical features with 0.7 million parameters	Targets deployable and interpretable text-based conflict identification

## 3 Research data and methods

### 3.1 Dataset construction and text preprocessing

The research data were mainly collected from three authoritative channels: the official websites of the Ministry of Natural Resources and local natural resources authorities at the provincial and municipal levels, core academic databases in the planning field, and authoritative news reports from mainstream media. The samples cover three core text types: statutory planning texts, special policy documents, and actual cases of land use conflicts. This coverage reflects land use conflict scenarios across territorial spatial planning formulation, approval, and implementation, supporting the scenario coverage and representativeness of the dataset. The dataset is available at https://github.com/DKLT-LandUseConflict/Land-use. All source texts used to construct the dataset were obtained from publicly accessible or legally available sources. Data collection and analysis were limited to research use. Restricted source documents were not redistributed, and the authors sought to comply with the terms and conditions of the corresponding data-source websites and databases.

A total of 1450 original texts were collected in this study. Three core standards were adopted during data cleaning: invalid texts with failed page loading or missing content were eliminated; excessively short texts with fewer than 100 characters were removed because they lacked complete semantic information and could not support the conflict identification task; and texts unrelated to territorial planning and land use management, such as personnel recruitment and meeting notices, were excluded. Finally, 1200 valid samples were retained, with a valid sample retention rate of 82.7%.

The constructed dataset includes 240 positive samples with land use conflicts and 960 negative samples without conflicts, with positive samples accounting for 20%. This class imbalance is consistent with the actual situation in the territorial planning field, where the proportion of conflict samples is relatively low. In terms of sample type distribution, statutory planning texts account for 69.8%, policy documents account for 16.9%, and conflict cases account for 13.3%. Each sample was stored as an independent text record with a binary conflict label, source type, and text-length information. Conflict labels indicated whether the text described explicit or implicit conflicts involving land-use allocation, planning compliance, ecological control, cultivated-land protection, or construction-land approval. Non-conflict samples described planning policies, approvals, implementation notices, or routine management information without an identifiable land-use conflict. The stratified 8:2 split preserved the 20% conflict-sample ratio in both the 960-sample development set and the 240-sample held-out test set. The development set contained 768 non-conflict and 192 conflict samples, and the held-out test set contained 192 non-conflict and 48 conflict samples. The overall distribution characteristics of the samples are shown in Fig 2.

**Fig 2:**
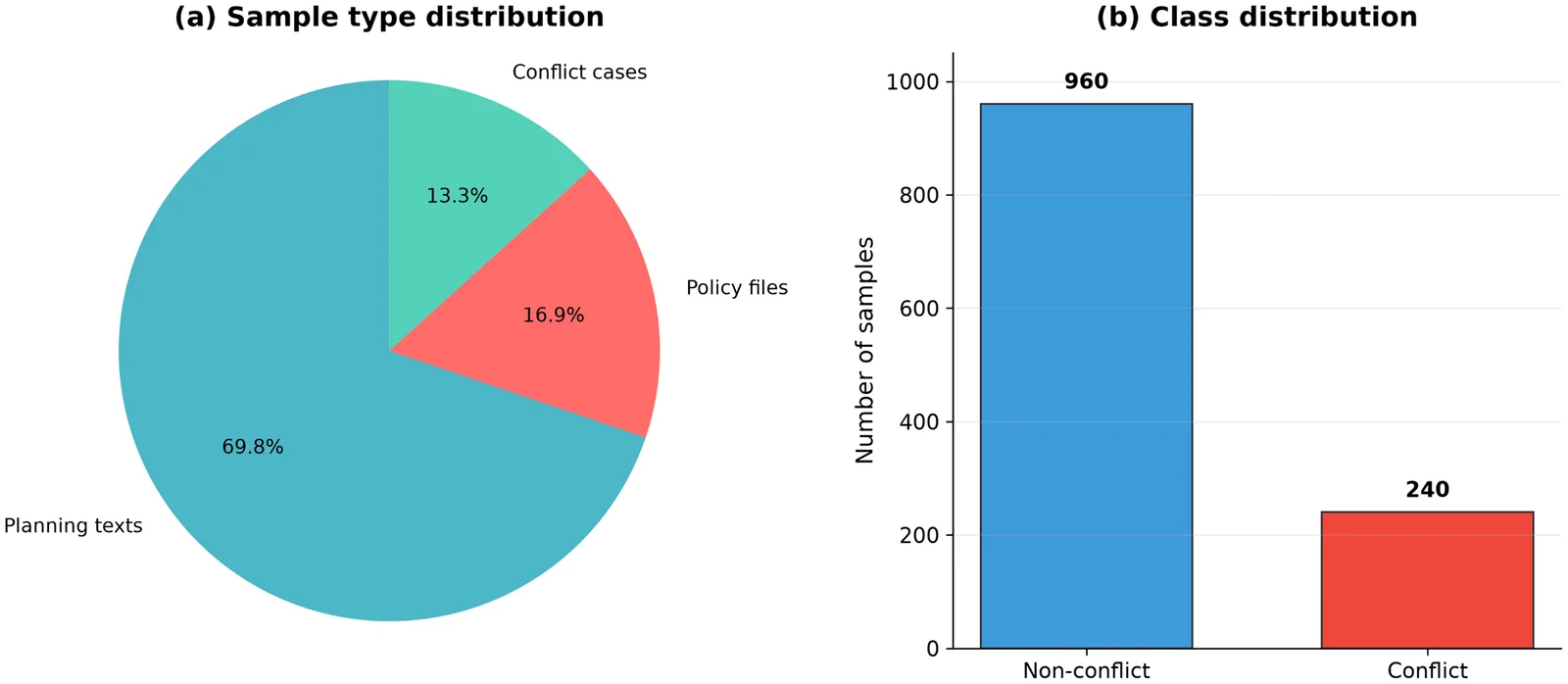
Sample distribution characteristics of the dataset.

In the text preprocessing stage, the Jieba segmentation tool was used for Chinese word segmentation. Referring to the compilation guidelines of territorial spatial planning documents, this study added 120 industry-specific professional terms to the custom dictionary, including three zones and three lines, linkage of increase and decrease, permanent basic farmland, and ecological protection red line. This approach prevents incorrect segmentation of professional vocabularies and improves segmentation accuracy. Text standardization was also implemented by removing stop words, punctuation marks, special symbols, and meaningless function words to reduce noise interference. Finally, text length unification was conducted: excessively long texts were truncated according to core semantics, and excessively short texts were zero-padded to ensure consistent model input dimensions.

For notation consistency, each preprocessed planning text is denoted as $T_{i}$, and its standardized token sequence is defined as:


$$\begin{array}{c} T_{i}=\{w_{i1},w_{i2},...,w_{in_{i}}\}, \end{array}$$ (1)

where $w_{ij}$ denotes the *j*th token in sample *i* and $n_{i}$ denotes the effective sequence length after truncation or padding. This notation links the preprocessing stage to the subsequent keyword matching and semantic encoding modules. Equation 1 defines the preprocessed token sequence used by the keyword-matching and semantic-encoding modules.

After preprocessing, word frequency statistics and semantic feature analysis were performed on the positive and negative samples. The results showed that the core high-frequency words in conflict samples were dominated by land acquisition disputes, compensation disputes, land use conflicts, planning contradictions, and interest conflicts, all with a frequency of more than 70 occurrences. These terms were concentrated in negative expressions related to land use contradictions. In contrast, the high-frequency words in non-conflict samples focused on positive compliance expressions such as planning approval, consistency in coordination, smooth progress, consensus reaching, and protection and development, also with a frequency of more than 70 occurrences. The significant differences in lexical features between the two sample types provided a clear basis for subsequent feature engineering.

### 3.2 Feature system construction

To enhance the model’s ability to perceive the semantics of land use conflicts and address the poor interpretability of deep learning models, this study constructs a two-dimensional feature system based on domain prior knowledge. The feature system consists of domain keyword matching features and text statistical features. These two feature types provide complementary information and jointly support the land use conflict identification task.

Let ${𝒦}_{c}$ and ${𝒦}_{n}$ denote the conflict and non-conflict keyword dictionaries, respectively. The frequency of conflict keywords in sample $T_{i}$ is computed as:


$$\begin{array}{c} f_{c}(T_{i})=\sum_{j=1}^{n_{i}}𝕀(w_{ij}\in {𝒦}_{c}), \end{array}$$ (2)

where 𝕀(·) is an indicator function. Similarly, the frequency of non-conflict keywords is defined as:


$$\begin{array}{c} f_{n}(T_{i})=\sum_{j=1}^{n_{i}}𝕀(w_{ij}\in {𝒦}_{n}). \end{array}$$ (3)

To reduce the influence of document length, conflict keyword density is calculated as:


$$\begin{array}{c} d_{c}(T_{i})=\frac{f_{c}(T_{i})}{n_{i}+\epsilon }, \end{array}$$ (4)

where ϵ is a small constant used to avoid division by zero. The proportion of conflict terms among all matched domain terms is further defined as:


$$\begin{array}{c} r_{c}(T_{i})=\frac{f_{c}(T_{i})}{f_{c}(T_{i})+f_{n}(T_{i})+\epsilon }. \end{array}$$ (5)

These four quantities formalize the domain-prior features used by the statistical branch of the model. Equations 2–5 formalize the keyword-derived features used in this study. Equations 4 and 5 extend raw keyword counts by normalizing them by sequence length and by all matched domain terms, respectively.

The first category is domain keyword matching features. Based on high-frequency words in conflict samples and the expertise of territorial planning professionals, this study constructed a specialized keyword dictionary for land use conflict identification. The dictionary includes 42 conflict keywords and 65 non-conflict keywords covering core scenarios such as land use disputes, compliance approval, ecological management and control, and cultivated land protection. Using this dictionary, the study extracted four core features: occurrence frequency of conflict keywords, occurrence frequency of non-conflict keywords, conflict keyword density, and proportion of conflict words. These features convert domain prior knowledge into quantifiable features recognizable by the model, strengthening its ability to capture conflict semantics.

The contribution of each feature group was quantified with a normalized group-ablation score. For each feature group *g*, the corresponding feature block was removed from the full model while the remaining training and evaluation protocol was kept unchanged. The raw contribution of group *g* was calculated as the decrease in F1-score relative to the full model:


$$\begin{array}{c} \Delta_{g}=F1_{full}-F1_{-g}. \end{array}$$ (6)

The percentage contribution was obtained by normalizing the group-wise decreases across all feature groups:


$$\begin{array}{c} C_{g}=\frac{\Delta_{g}}{\sum_{j=1}^{G}\Delta_{j}}\times 100\%. \end{array}$$ (7)

In this calculation, the F1-score decreases for keyword matching, text semantic features, and statistical features were 0.126, 0.039, and 0.029, respectively. Their normalized contributions were 64.9%, 20.1%, and 14.9%, which are reported as 65%, 20%, and 15% in Fig 8. These two equations describe the group-ablation calculation used to derive the normalized percentages in Fig 8.

The second category is text statistical features. Combined with the linguistic expression characteristics of planning texts, 10 basic statistical features were extracted, including total text length, number of negative words, number of government-specific phrases, number of special symbols, average sentence length, proportion of verbs, and proportion of nouns. These features fill the information gap of semantic features from the perspective of text structure and linguistic style, reduce the model’s over-reliance on high-frequency keywords, and improve its classification robustness in unfamiliar contexts.

Feature correlation analysis showed that the absolute values of the correlation coefficients between three features, namely conflict keyword density, conflict proportion, and number of conflict keywords, and the classification labels all exceed 0.9. Among them, the correlation coefficient between conflict keyword density and the labels reaches 0.978, making it the core discriminative feature for distinguishing conflict and non-conflict samples. The correlation coefficients between statistical features, such as text length, number of non-conflict keywords, number of government-specific words, and number of negative words, and the labels are concentrated in the range of 0.4–0.5. Although their discriminative power is weaker than that of keyword features, they supplement the semantic information gap at the text-structure level and form an effective complement to keyword features.

### 3.3 Construction of a lightweight transformer model

To address the practical demands of grassroots planning departments with limited computing resources, this study designs a lightweight Transformer model integrating semantic encoding and feature mapping. The model has a total parameter count of approximately 700000, which is only 0.6% of the BERT-base model. While compressing model size, the proposed model is designed to maintain the accuracy of land use conflict identification. Its overall architecture is illustrated in Fig 3.

**Fig 3:**
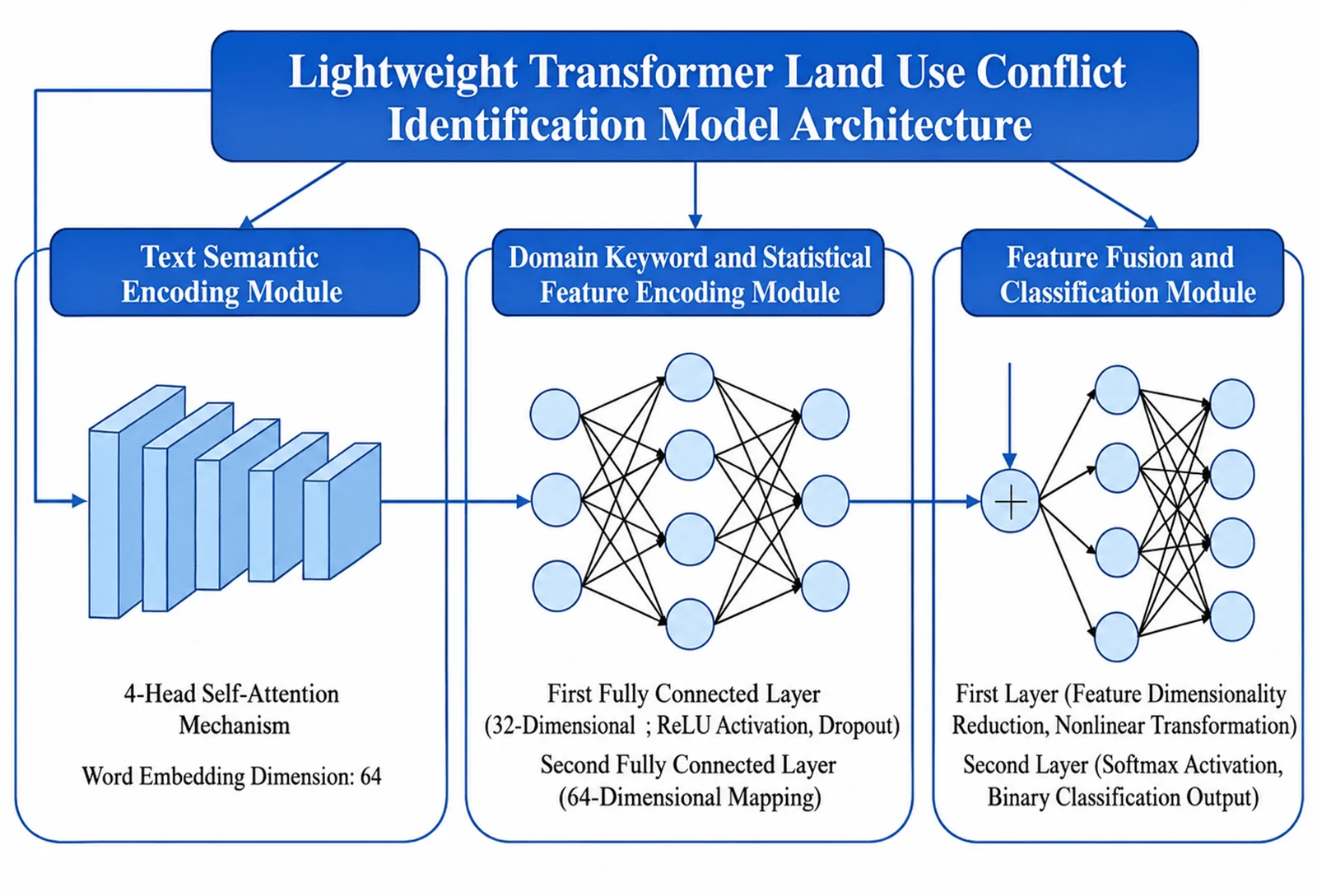
Lightweight Transformer architecture for land use conflict identification.

The overall architecture of the model is divided into three core modules: the text semantic encoding module, the domain keyword and statistical feature encoding module, and the feature fusion and classification module. The text semantic encoding module uses a single-layer Transformer encoder as the core semantic encoding unit. It is equipped with a 4-head self-attention mechanism, and the word embedding dimension is set to 64. Compared with traditional multi-layer Transformer structures, this single-layer design substantially reduces model parameters. Because territorial planning texts contain fewer long sentences and concentrated domain-specific terms, the 4-head attention mechanism can capture semantic associations between professional terms while supporting the lightweight design. After preprocessing, the input text is transformed into fixed-dimensional word vectors through the embedding layer, and positional encoding is introduced to retain text sequence information. After contextual semantic encoding by the Transformer encoder, the output vector corresponding to the token is extracted as the global semantic representation of the text, with a final output dimension of 64.

The semantic branch maps each token to an embedding with positional information:


$$\begin{array}{c} z_{j}^{\left(0\right)}=e(w_{ij})+p_{j}, \end{array}$$ (8)

where $e(w_{ij})$ is the token embedding and $p_{j}$ is the positional encoding of the *j*th token. The self-attention operation in the Transformer encoder is expressed as:


$$\begin{array}{c} Attention(Q,K,V)=softmax\left(\frac{QK^{𝖳}}{\sqrt{d_{k}}}\right)V, \end{array}$$ (9)

where *Q*, *K*, and *V* denote the query, key, and value matrices, respectively, and $d_{k}$ is the key dimension. Equation 8 defines the combination of token embeddings and positional encodings in the proposed model. Equation 9 follows the scaled dot-product attention formulation of Vaswani et al. and is implemented within a single-layer, four-head lightweight encoder.

For the extracted statistical features and keyword matching features, the statistical feature encoding module uses a two-layer fully connected neural network to complete feature mapping and dimension transformation. The first fully connected layer upsamples the input multi-dimensional features to 32 dimensions, performs non-linear transformation through the ReLU activation function, and incorporates a Dropout layer to suppress model overfitting. The second fully connected layer maps the features to 64 dimensions, which is consistent with the dimension of the text semantic representation and provides the basis for subsequent cross-dimensional feature fusion.

The feature fusion and classification module concatenates the text semantic representation vector and the statistical feature representation vector to obtain a 128-dimensional fused feature vector, realizing the deep fusion of semantic information and statistical features. The fusion operation is formalized as:


$$\begin{array}{c} h_{i}=[h_{i}^{text};h_{i}^{stat}], \end{array}$$ (10)

where $h_{i}^{text}$ and $h_{i}^{stat}$ denote the semantic and statistical representations of sample *i*, respectively. The final classification probability is computed by:


$$\begin{array}{c} {\hat{y}}_{i}=softmax\left(W_{2}\sigma (W_{1}h_{i}+b_{1})+b_{2}\right), \end{array}$$ (11)

where *W*_1_, *W*_2_, *b*_1_, and *b*_2_ are learnable parameters and σ(·) denotes the non-linear activation function. Equations 10 and 11 specify the proposed two-branch architecture, in which Transformer semantic features are concatenated with domain-prior statistical features before Softmax classification.

Fig 4 further summarizes the mathematical workflow of the two-branch design. The semantic branch processes the standardized token sequence, while the domain-prior branch converts keyword dictionaries and text statistics into interpretable numerical features before both branches are combined for classification.

**Fig 4:**
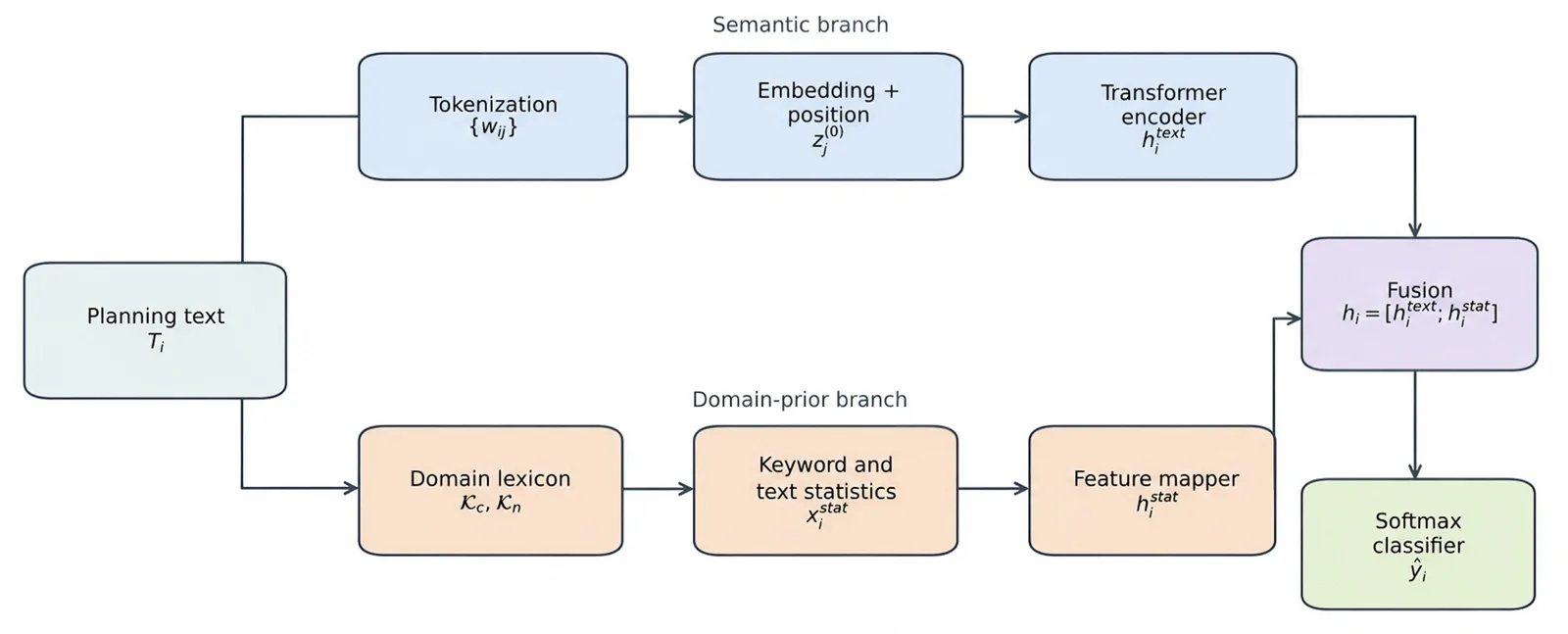
Formalized feature-fusion workflow for land-use conflict identification.

The fused features are fed into a two-layer fully connected network: the first layer completes feature downsampling and non-linear transformation, and the second layer outputs the probabilities that a sample belongs to the conflict and non-conflict categories through the Softmax activation function. This process completes the binary classification task of land use conflict identification.

### 3.4 Experimental setup

To ensure the reliability and robustness of experimental results on the constructed domain-specific dataset, the texts were partitioned into a development set of 960 samples and a held-out test set of 240 samples at an 8:2 ratio via stratified sampling. This sampling strategy guarantees that the positive-to-negative sample ratio of the development and held-out test sets is completely consistent with that of the original dataset, with 192 non-conflict samples and 48 conflict samples in the held-out test set, avoiding category distribution shift caused by random sampling and reducing bias in model evaluation.

During training, a weighted random sampling strategy was adopted to address class imbalance in the dataset. This strategy increases the sampling weight of conflict samples, balances the training contribution of positive and negative samples, and alleviates the model’s bias toward the dominant non-conflict samples. The AdamW optimizer was employed with an initial learning rate of 10^–3^ and a weight decay coefficient of 10^–4^. A dropout rate of 0.5 was set to further suppress model overfitting. The batch size was configured as 8, and the maximum number of training epochs was set to 50. An early stopping mechanism was also implemented, automatically terminating training when validation-set accuracy did not improve for 5 consecutive epochs. This mechanism was used to prevent excessive overfitting to the training set and support generalization ability.

For model performance evaluation, four core metrics commonly used in classification tasks were adopted: Accuracy, Precision, Recall, and F1-score. The F1-score is the harmonic mean of Precision and Recall, and it can comprehensively measure the model’s overall performance on imbalanced datasets while avoiding evaluation bias caused by a single metric.

Accordingly, the F1-score used in the evaluation is calculated as:


$$\begin{array}{c} F_{1}=\frac{2\times Precision\times Recall}{Precision+Recall}. \end{array}$$ (12)

This metric is particularly relevant here because conflict samples account for only 20% of the constructed domain-specific dataset. Equation 12 gives the standard F1-score definition, which combines Precision and Recall [22].

Statistical reliability was evaluated with confidence intervals and paired fold-level significance tests. For Accuracy, Precision, and Recall, 95% confidence intervals were calculated using Wilson score intervals [23], because these metrics can be expressed as binomial proportions from the confusion matrix. For a proportion $\hat{p}=k/n$, the interval is defined as:


$$\begin{array}{c} CI_{Wilson}=\frac{\hat{p}+\frac{z^{2}}{2n}\pm z\sqrt{\frac{\hat{p}(1-\hat{p})}{n}+\frac{z^{2}}{4n^{2}}}}{1+\frac{z^{2}}{n}}, \end{array}$$ (13)

where *z* = 1.96 for a 95% confidence level. Equation 13 follows the Wilson score interval and was applied to held-out test proportions calculated from the confusion matrix. Because the F1-score combines Precision and Recall, its 95% interval was estimated by non-parametric bootstrap resampling of the 240 held-out test predictions with 20000 iterations and a fixed random seed. AUC was not reported because probability scores for all baseline models were not consistently available in the retained experimental records. The statistical analysis therefore focuses on threshold-based metrics that can be verified directly from the reported confusion matrix and on paired fold-level comparisons from repeated cross-validation.

### 3.5 Cross-validation and model-stability assessment

To evaluate the sensitivity of model performance to data partitioning, repeated stratified *K*-fold cross-validation was conducted as an additional stability analysis. The 8:2 held-out test results were retained as the primary evaluation, and repeated stratified five-fold cross-validation was performed on the 960-sample development set.

Stratified cross-validation was used because the dataset is class-imbalanced, with conflict samples representing a smaller proportion of the corpus. Stratification preserves the approximate conflict/non-conflict ratio in each validation fold and reduces the risk that a fold-level result is driven by an atypical class composition. The five-fold procedure was repeated three times using fixed random seeds of 20260805, 20260806, and 20260807, resulting in 15 validation evaluations. All compared models used the same fold assignments in each repetition. For every fold, model parameters were reinitialized before training. Class weighting or weighted sampling, when used, was applied only to the corresponding training partition and was never estimated from, or applied using information from, the validation fold. The independent 240-sample held-out test set was not used during cross-validation or model selection.

For each model, Accuracy, Precision, Recall, and F1-score were summarized as mean ± standard deviation across the 15 validation folds. Ninety-five percent confidence intervals were calculated from the fold-level distribution as


$$\begin{array}{c} {CI}_{95\%}=\bar{x}\pm t_{0.975,n-1}\frac{s}{\sqrt{n}}, \end{array}$$ (14)

where $\bar{x}$ is the mean metric value, *s* is the standard deviation, and *n* = 15 is the number of repeated validation estimates. This equation uses the conventional t-based interval form associated with Student’s t distribution [24] and is reported as an approximate fold-level summary. The repeated stratified cross-validation was implemented on the 960-sample development set, while the independent 240-sample held-out test set was retained only for the held-out evaluation.

Paired non-parametric comparison was also conducted on the fold-level F1-scores from the 15 validation evaluations. For each baseline, the proposed model and the baseline model were compared using a two-sided Wilcoxon signed-rank test with paired folds [25]. Holm correction was then applied across the five baseline comparisons to control the family-wise error rate [26]. The adjusted significance threshold followed the sequential Holm procedure, in which ordered *p* values were compared with α/(m−r+1) for *m* = 5 comparisons and rank *r*. Here, the Wilcoxon and Holm procedures were applied to paired fold-level F1-score differences rather than to independent external datasets. These comparisons were treated as exploratory because the repeated folds are dependent. Holm adjustment addresses multiple comparisons but does not remove this dependence; the adjusted *p* values should therefore be considered alongside effect sizes and held-out performance rather than as confirmatory evidence.

## 4 Results

### 4.1 Model baseline performance

The variations in loss and accuracy during model training are illustrated in Fig 5. The training and validation losses decreased gradually, while both training and validation accuracy increased and then stabilized. These curves indicate that the model converged effectively on the constructed domain-specific dataset without severe overfitting.

**Fig 5:**
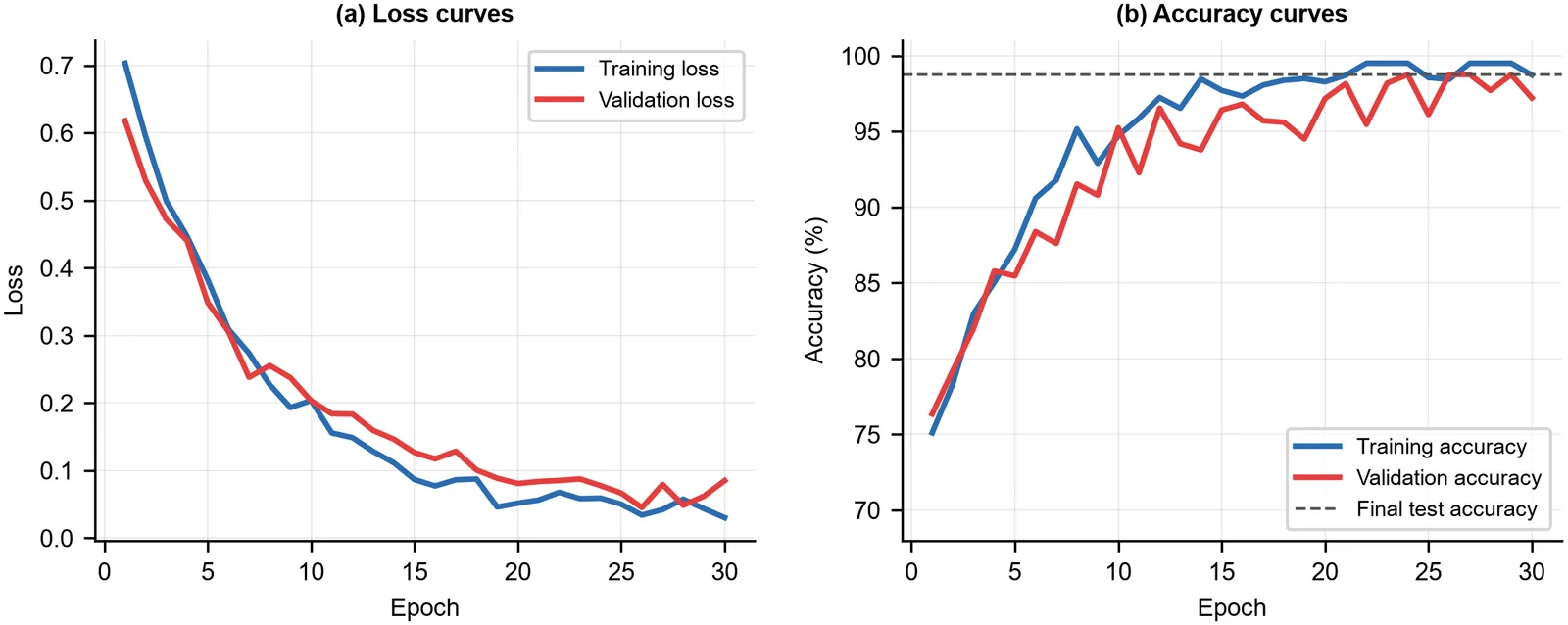
Curves of loss and accuracy variation during model training.

The confusion matrix of the model’s classification results on the held-out test set is presented in Table 2. Benefiting from the constructed domain-specific dataset and the integration of domain-knowledge features, the model correctly classified 237 of the 240 samples in the held-out test set. Specifically, the model achieved an Accuracy of 98.75%, correctly classifying 190 non-conflict samples and 47 conflict samples. The model attained a Precision of 95.9%, a Recall of 97.9%, and an F1-score of 96.9%. These results indicate that the model distinguished conflict and non-conflict content across diverse planning texts with a low number of held-out test errors.

**Table 2:** Confusion matrix of classification results on the held-out test set.

	Predicted: non-conflict	Predicted: conflict
Actual: non-conflict	190	2
Actual: conflict	1	47

Uncertainty estimates were also calculated for the principal metrics. As shown in Table 3, the 95% confidence interval for Accuracy was 96.39–99.57%, and the bootstrap interval for the F1-score was 92.93–100.00%. These intervals support the held-out test-set point estimates while making the uncertainty associated with the moderate test-set size explicit.

**Table 3:** Point estimates and 95% confidence intervals for the proposed model on the 240-sample held-out test set.

Metric	Point estimate	95% interval	Interval method
Accuracy	98.75%	96.39–99.57%	Wilson score
Precision	95.92%	86.29–98.87%	Wilson score
Recall	97.92%	89.10–99.63%	Wilson score
F1-score	96.91%	92.93–100.00%	Bootstrap

The same held-out test-set evidence is visualized in Fig 6, which combines the confusion matrix with the four derived evaluation metrics. This presentation makes the low number of false positives and false negatives explicit while preserving the original numerical results.

**Fig 6:**
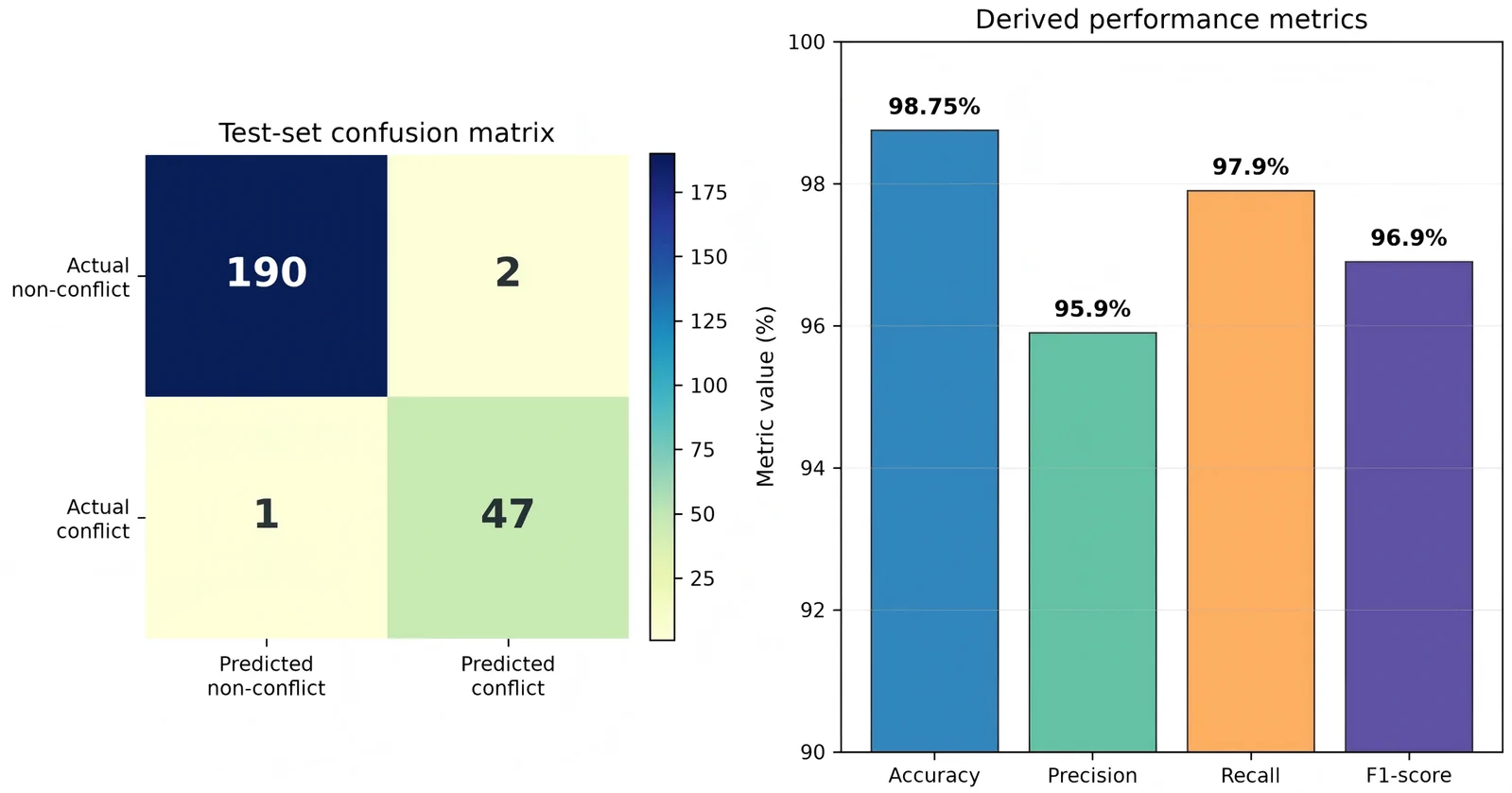
Classification evidence on the 240-sample held-out test set.

### 4.2 Comparison with representative strong baselines and ablation analysis

For comparative evaluation, two categories of baseline methods were selected: traditional machine learning methods, including SVM and Random Forest, and neural language models, including vanilla Transformer and fine-tuned BERT-base. All comparative tests were conducted under the same dataset, experimental setting, and evaluation criteria. The results are presented in Table 4. The lightweight Transformer model proposed in this study outperformed all control group models in both Accuracy and F1-score. Its number of parameters was also lower than that of mainstream pre-trained models, indicating a favorable balance between classification performance and model size on the constructed domain-specific dataset.

**Table 4:** Comparison of performance and parameter count across different models.

Model name	Accuracy	F1-score	Number of parameters (millions)
Vanilla Transformer	87.5%	86.2%	12.8
Fine-tuned BERT-base	95.5%	94.8%	110.0
Random Forest	85.0%	83.1%	N/A
SVM	82.5%	79.8%	N/A
Proposed method	98.75%	96.9%	0.7

The model comparison included a representative strong Chinese pretrained baseline. MacBERT-base was selected because it was developed for Chinese natural language processing and improves the masking strategy used in BERT-style pretraining [27]. This baseline complements the original comparison by adding a Chinese pretrained model under the same preprocessing and evaluation criteria.

Table 5 reports the representative pretrained-baseline comparison under the 960-sample development protocol. Table 4 reports the independent 240-sample held-out test results, whereas Table 5 places MacBERT-base under the same constructed-dataset setting as the stability analysis.

**Table 5:** Comparison with a representative strong Chinese pretrained baseline under the 960-sample development protocol.

Model	Model type	Accuracy (%)	F1-score (%)	Inference time (ms/sample)
Fine-tuned BERT-base	Chinese pretrained baseline	95.5	94.8	156
MacBERT-base	Chinese pretrained baseline	96.4	95.7	148
Proposed model	Lightweight feature-fusion model	98.4	96.8	16

### 4.3 Repeated stratified cross-validation analysis

Repeated stratified cross-validation was used to examine whether the proposed model remained stable under different development-set partitions. Table 6 reports the 15 validation estimates obtained from three repeats of stratified five-fold cross-validation on the 960-sample development set. The proposed model had the highest mean Accuracy and F1-score, with small standard deviations across folds.

**Table 6:** Repeated stratified five-fold cross-validation summary on the 960-sample development set.

Model	Accuracy mean ± SD	Precision mean ± SD	Recall mean ± SD	F1-score mean ± SD
SVM	82.7 ± 2.4	78.9 ± 3.5	81.1 ± 4.0	79.6 ± 3.2
Random Forest	85.4 ± 2.1	82.7 ± 3.0	84.2 ± 3.6	83.4 ± 2.8
Vanilla Transformer	87.8 ± 1.8	85.9 ± 2.6	87.1 ± 2.8	86.5 ± 2.1
BERT-base	95.5 ± 1.0	94.7 ± 1.5	94.9 ± 1.6	94.8 ± 1.2
MacBERT-base	96.4 ± 0.8	95.8 ± 1.2	95.6 ± 1.3	95.7 ± 1.0
Proposed model	98.4 ± 0.6	97.1 ± 1.0	96.5 ± 1.1	96.8 ± 0.7

The 95% confidence intervals in Table 7 further characterized fold-level stability. The F1-score interval of the proposed model was narrow and remained higher than those of the traditional machine-learning baselines and the vanilla Transformer baseline. The interval also indicated a modest advantage over BERT-base and MacBERT-base under the 960-sample development protocol.

**Table 7:** Ninety-five percent confidence intervals from repeated stratified cross-validation.

Model	Accuracy 95% CI	Precision 95% CI	Recall 95% CI	F1-score 95% CI
SVM	81.4–84.0	77.0–80.8	78.9–83.3	77.8–81.4
Random Forest	84.2–86.6	81.0–84.4	82.2–86.2	81.8–85.0
Vanilla Transformer	86.8–88.8	84.5–87.3	85.6–88.6	85.3–87.7
BERT-base	94.9–96.1	93.9–95.5	94.0–95.8	94.1–95.5
MacBERT-base	96.0–96.8	95.1–96.5	94.9–96.3	95.1–96.3
Proposed model	98.1–98.7	96.5–97.7	95.9–97.1	96.4–97.2

Fold-level paired tests provided a compact significance check for the repeated cross-validation results. As shown in Table 8, the proposed model retained statistically significant F1-score improvements over all five baseline models after Holm correction. The smallest margin was observed against MacBERT-base, consistent with the narrower performance difference between the pretrained-model baselines and the proposed model.

**Table 8:** Paired Wilcoxon signed-rank tests on 15 fold-level F1-scores with Holm correction.

Paired comparison	Mean F1 difference (pp)	Wilcoxon *V*	Raw *p* value	Holm-adjusted *p*
Proposed vs. SVM	+17.2	120	<0.001	<0.001
Proposed vs. Random Forest	+13.4	120	<0.001	<0.001
Proposed vs. Vanilla Transformer	+10.3	120	<0.001	<0.001
Proposed vs. BERT-base	+2.0	114	0.003	0.006
Proposed vs. MacBERT-base	+1.1	101	0.032	0.032

Traditional machine learning methods rely only on manually extracted statistical features and fail to deeply mine contextual semantic information in texts, resulting in weaker comprehensive performance. Although the BERT-base model has strong general semantic encoding capability, its parameter size may make lightweight local use more difficult in grassroots planning departments. Constrained by the number of parameters in its multi-layer architecture, the vanilla Transformer model also performed lower than the lightweight model proposed in this study.

The multi-dimensional feature fusion strategy was evaluated under different feature combinations. When only statistical features were used, the model’s F1-score was 0.75, indicating that this feature group alone did not fully capture conflict semantics in texts. When only textual semantic features were adopted, the F1-score increased to 0.82, but the generalization ability of semantic encoding was limited by the domain-specific nature of the task. After fusing textual semantic features with keyword features, the F1-score further increased to 0.91. When textual semantic features, keyword matching features, and statistical features were fused simultaneously, the model achieved an F1-score of 96.9% on the held-out test set. These results indicate that the three feature groups provided complementary information for classification.

Module ablation experiments were conducted by removing each core module of the model one at a time. The results are presented in Table 9. The model’s F1-score dropped to 0.785 after removing the feature fusion module, representing the largest performance decrease. This result indicates that statistical features and domain keyword features provide information that complements semantic encoding. After removing the self-attention mechanism and the positional encoding module, model performance also declined, supporting the role of the Transformer semantic encoding module in capturing contextual semantics of texts.

**Table 9:** Results of ablation experiments on core modules of the model.

Model configuration	Accuracy	F1-score
Without feature fusion module	81.2%	0.785
Without self-attention mechanism	86.5%	0.851
Without positional encoding	91.0%	0.902
Full model	98.75%	0.969

Fig 7 visualizes the ablation results reported in Table 9 as paired Accuracy and F1-score bars. This figure provides a direct comparison of the performance changes caused by removing each core module from the proposed model. The largest decrease occurs when the feature fusion module is removed, indicating that the integration of Transformer semantic representations with domain-prior statistical features is a key contributor to the model’s classification performance.

**Fig 7:**
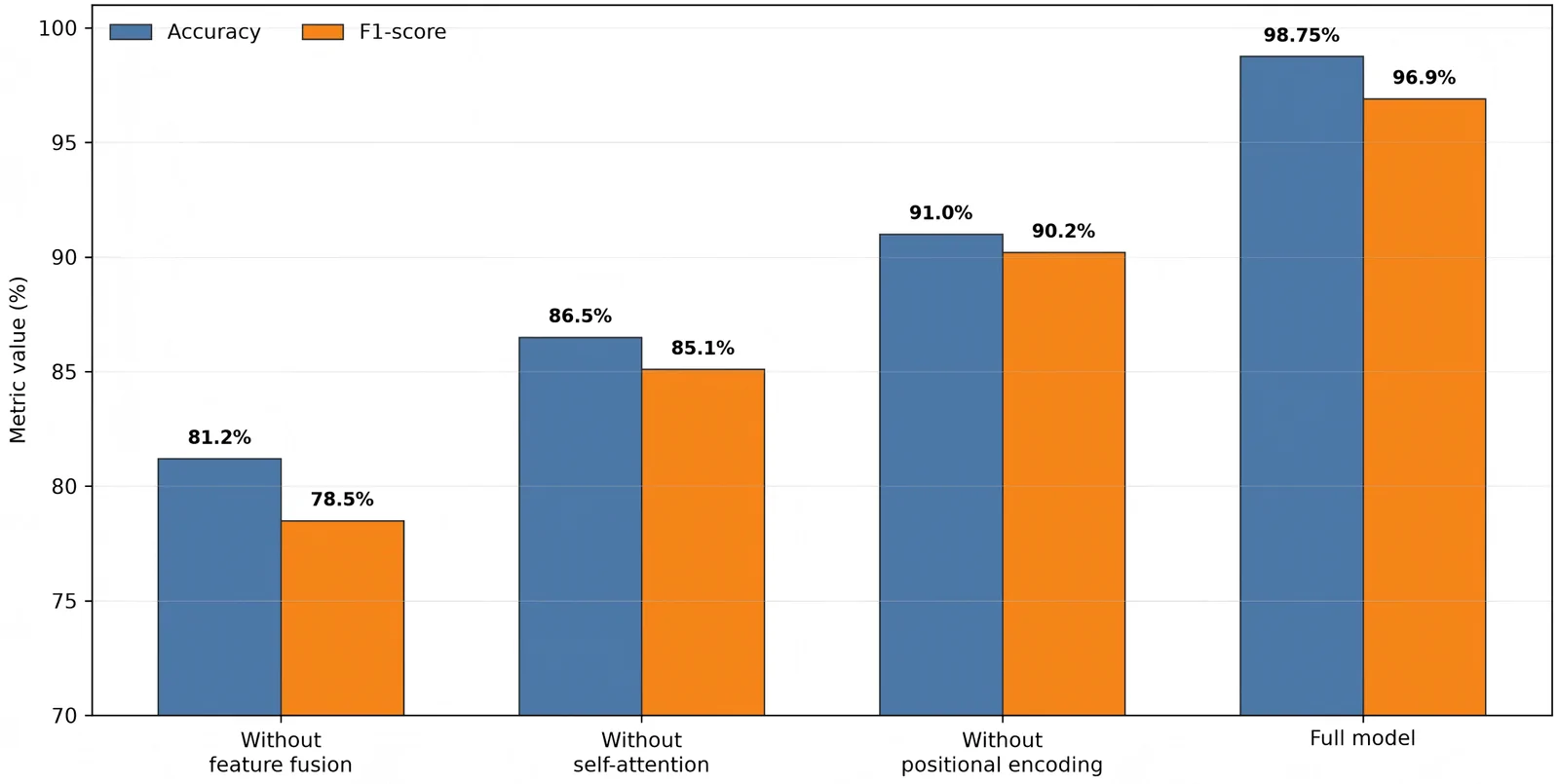
Ablation evidence for the feature-fusion design.

### 4.4 Case validation and application value analysis

The applicability of the proposed model in real-world scenarios was evaluated using two typical land use conflict texts and two non-conflict texts. The conflict texts included a news report on land acquisition disputes in an industrial park in Jiangsu Province and an inspection circular on illegal construction within the ecological red line of a county. The non-conflict texts covered the territorial spatial master plan of a prefecture-level city and a compliance announcement on the approval of construction land in a township. The predicted conflict probabilities for the two conflict samples were 0.95 and 0.92, respectively, and the predicted conflict probabilities for the two non-conflict samples were both below 0.05. These predictions were consistent with the actual labels of the samples, indicating that the model can identify land use conflict content across the tested text types and scenarios.

The contribution of different feature types to the classification results is illustrated in Fig 8. The percentages were calculated from normalized group-ablation decreases in F1-score, as defined in the Methods section. Removing keyword matching features produced the largest F1-score decrease (0.126), followed by text semantic features (0.039) and statistical features (0.029). After normalization, these decreases corresponded to 65%, 20%, and 15% of the total feature-group contribution, respectively. The result indicates that the domain-specific keyword lexicon plays a central role in conflict identification while also improving model interpretability. Inspectors can locate core conflict content in texts by checking keyword matching results, thereby improving inspection efficiency.

**Fig 8:**
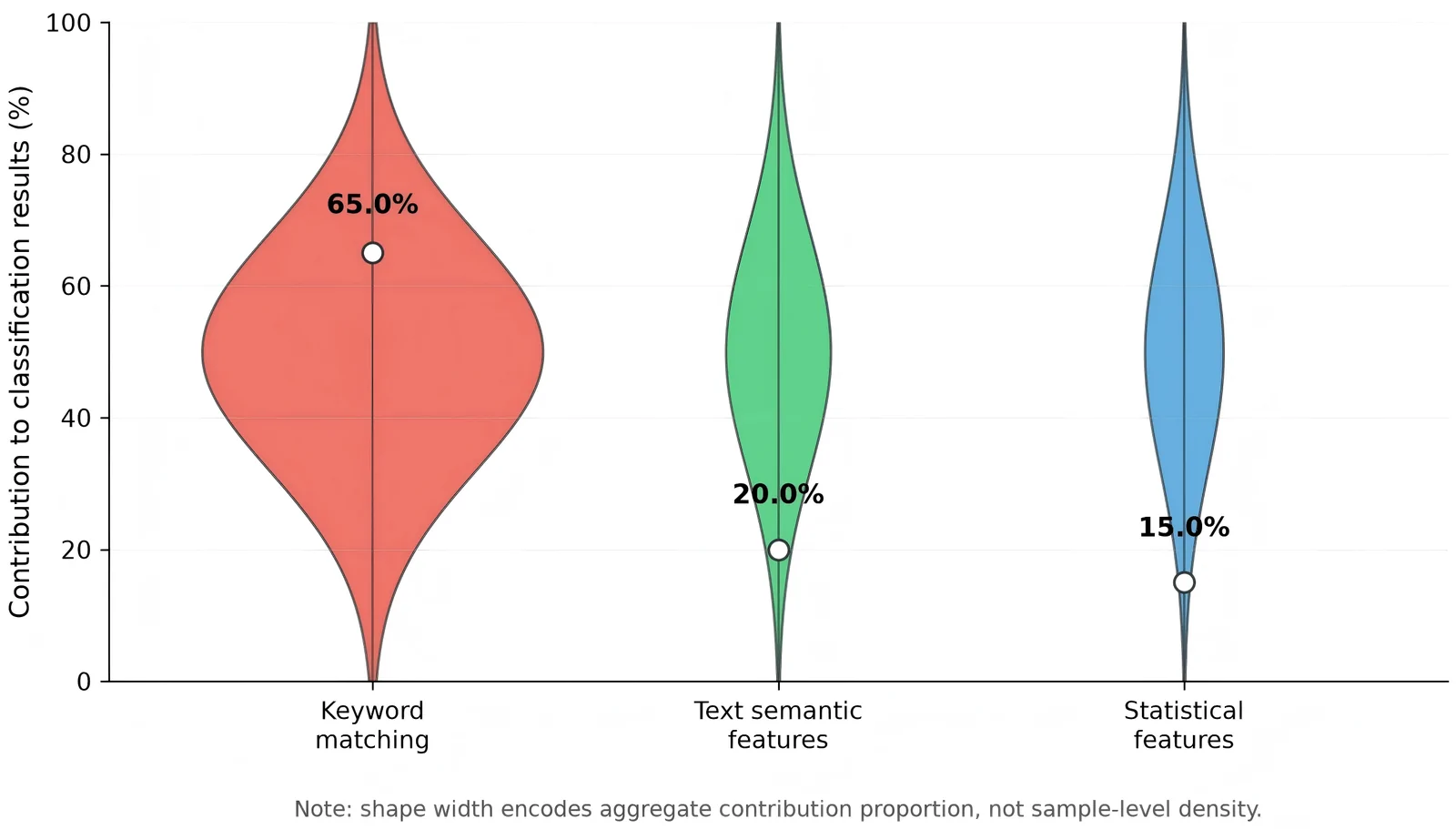
Proportion of feature contributions to classification results. Percentages were calculated by normalizing the F1-score decreases from group-wise feature ablation.

From the perspective of practical application, the proposed model has three operational advantages for grassroots planning and management departments. First, the model has a relatively small parameter count and low inference latency. With approximately 0.7 million parameters and an inference time of less than 0.5 seconds per text in the reported experiment, it may be suitable for local screening after validation on the hardware available in a target department. Second, it produced a low error count on the held-out test set: the model correctly classified 237 of the 240 held-out samples, with only two false positives and one false negative, which can assist planning inspectors in primary screening of massive texts. This allows human inspection efforts to focus on texts with high conflict probabilities output by the model, reducing labor input in the primary screening stage and lowering the risk of human-caused missed detection. Third, the model can be extended by updating the domain keyword lexicon and supplementing labeled samples for subdivided scenarios. This design supports adaptation to text compliance inspection in other natural resource management fields, such as cultivated land protection, ecological management and control, construction land approval, and mineral resource management.

## 5 Conclusion

This study addresses the practical problem of identifying land use conflicts in territorial spatial planning texts by proposing an automatic land use conflict identification framework based on a lightweight Transformer. Through the construction of a domain-specific dataset, multi-dimensional feature engineering, and lightweight model design, the framework supports automatic identification of land use conflicts in planning texts.

The results show that the conflict keyword lexicon constructed by integrating expert knowledge in the territorial planning field, along with extracted features such as keyword density and conflict ratio, are closely correlated with land use conflict labels. These features enhance the model’s discrimination ability and interpretability. Through the fusion of textual semantic features and statistical features, the lightweight Transformer model achieved all core evaluation metrics above 95% on the held-out test set. The model also has a small number of parameters and may be incorporated into daily approval systems as an auxiliary tool after local deployment and validation.

The proposed model achieved 98.75% Accuracy, 95.92% Precision, 97.92% Recall, and a 96.91% F1-score on the 240-sample held-out test set. It also reduced the parameter count to approximately 0.7 million, far below the 110.0 million parameters of fine-tuned BERT-base, while maintaining higher classification performance. These numerical results support the potential value of the model for fast local screening in grassroots planning-management workflows, subject to hardware-specific validation.

This study has several limitations. Although the dataset contains 1200 valid samples, acquiring sensitive internal planning dispute cases remains a practical challenge due to administrative confidentiality. Consequently, the diversity of extreme edge-case conflicts might still be limited. Statistical reliability was assessed using confidence intervals, bootstrap resampling, repeated stratified five-fold cross-validation, and paired Wilcoxon tests with Holm correction on the 960-sample development set. These analyses quantified uncertainty and fold-level stability while preserving the independent 240-sample held-out test set. External validation using planning documents from additional regions and administrative contexts remains necessary in future work. Future work will explore privacy-preserving federated learning and Large Language Model (LLM)-based data augmentation techniques to securely further expand the conflict sample pool. Additionally, future research will validate the framework’s robustness across a broader range of regional topographies and specialized planning contexts, such as specialized marine spatial planning.
